# Colostrum supplementation protects against exercise - induced oxidative stress in skeletal muscle in mice

**DOI:** 10.1186/1756-0500-5-649

**Published:** 2012-11-22

**Authors:** Mahenderan Appukutty, Ammu Kutty Radhakrishnan, Kalavathy Ramasamy, Rajesh Ramasamy, Abu Bakar Abdul Majeed, Mohd Ismail Noor, Nik Shanita Safii, Poh Bee Koon, Karuthan Chinna, Nagaraja Haleagrahara

**Affiliations:** 1 Faculty of Sports Science & Recreation, University Technology Mara, Shah Alam 40450, Kuala Lumpur, Malaysia; 2 Faculty of Medicine and Health, International Medical University, Bukit Jalil 57000, Kuala Lumpur, Malaysia; 3 Faculty of Pharmacy, University Technology Mara, Shah Alam 40450, Kuala Lumpur, Malaysia; 4 Faculty of Medicine and Health Sciences, University Putra Malaysia, Serdang 43400, Kuala Lumpur, Malaysia; 5 Faculty of Health Sciences, University Technology Mara, Shah Alam 40450, Kuala Lumpur, Malaysia; 6 Faculty of Health Sciences, Universiti Kebangsaan Malaysia, Bangi 43600, Kuala Lumpur, Malaysia; 7 Faculty of Medicine, University Malaya 50603, Kuala Lumpur, Malaysia; 8 Nagaraja Haleagrahara, School of Veterinary and Biomedical Sciences, Faculty of Medicine, Health and Molecular Sciences, James Cook University, Townsville 4811, QLD, Australia

**Keywords:** Bovine colostrum, Exercise, Skeletal muscle, Antioxidants, Lipid hydroperoxides

## Abstract

**Background:**

This study examined the effects of bovine colostrum on exercise –induced modulation of antioxidant parameters in skeletal muscle in mice. Adult male BALB/c mice were randomly divided into four groups (control, colostrum alone, exercise and exercise with colostrum) and each group had three subgroups (day 0, 21 and 42). Colostrum groups of mice were given a daily oral supplement of 50 mg/kg body weight of bovine colostrum and the exercise group of mice were made to exercise on the treadmill for 30 minutes per day. Total antioxidants, lipid hydroperoxides, xanthine oxidase and super oxide dismutase level was assayed from the homogenate of hind limb skeletal muscle.

**Results:**

Exercise—induced a significant oxidative stress in skeletal muscles as evidenced by the elevated lipid hydroperoxides and xanthine oxidase levels. There was a significant decrease in skeletal muscle total antioxidants and superoxide dismutase levels. Daily colostrum supplement significantly reduced the lipid hydroperoxides and xanthine oxidase enzyme level and increased the total antioxidant levels in the leg muscle.

**Conclusion:**

Thus, the findings of this study showed that daily bovine colostrum supplementation was beneficial to skeletal muscle to reduce the oxidant-induced damage during muscular exercise.

## Background

Bovine colostrum [BC] has been used in therapeutic medicine for years by many cultures around the world. Colostrum is the first nutrition that newborn receive after birth. Bovine colostrum is a protein-rich cocktail that contains several nutritional and immunological factors that provide a strong nutritional base for the newborn animal [1,2]. BC is currently promoted as a supplement in sports nutrition for muscle recovery, anaerobic sports functions and also to some extent for its anti-aging property [3,4]. Several studies have reported the use of BC for immune responses and sporting performance with doses ranging from 20 to 60 g per day [5-7] and the recent study has reported a dose as low as 10 g per day that have proven to have a significant effect on human trial [8].

One of the earliest studies that investigated the effect of BC supplementation on sporting performance in humans showed that eight days of low-dose BC supplementation during speed and strength training did not have any effect on vertical jump or recovery from exercise [9]. However, later studies have shown that eight-weeks of BC supplementation of 20 g per day during endurance and resistance training significantly increased lean body mass, but no effect was observed on bench press performance [10]; while daily supplementation of 60 g BC per day for eight-weeks significantly improved sprint ability and indicated a trend towards improved vertical jump test performance [11].

Reactive oxygen species [ROS] represent a broad spectrum of species including non-radical derivatives of oxygen (hydrogen peroxide) that are also capable of inciting oxidative tissue damage [12]. Antioxidants are substances that help to reduce the severity of oxidative stress. Antioxidants may neutralize the reactive species, which are produced by neutrophils during phagocytosis [12,13]. Antioxidant and anti-inflammatory supplements have been promoted to decrease oxidative stress and inflammation due to physiological stressors such as exercise [14]. To date, the question of exercise-induced oxidative stress had brought about a lot of investigations, but none could give the precise adaptation of antioxidants. Margaritis and Rousseau [15] in an intensive review noted that whether acute or chronic physical exercise induces a change in antioxidants has not been sufficiently elucidated. Most studies on antioxidants and exercise, focused on micronutrients and as of date, the effect of bovine colostrum and physical exercise has not been discussed in the literature. Thus, the present investigation was conducted to determine the effect of colostrum supplementation on exercise – induced modulation of antioxidant enzymes and lipid peroxidation in skeletal muscles of mice. The hypothesis of the study was that the colostrum supplementation will reduce exercise-induced severe oxidative damage in skeletal muscle in mice.

## Results and discussion

The body weight and food intake changes were not shown as there was no statistically significant weight change and food and water intake difference throughout the duration of the study.

### Total proteins

The mean values for total proteins found in the muscle homogenate from mice in the various groups over time are shown in Table 1. Results of one way analysis of the data at baseline showed that day 21 group had significantly higher [p < 0.05] total proteins than control 21 day group. However, protein concentrations increased significantly after 42 days [p < 0.05] in all the three experimental groups and this increase was significantly higher [p < 0.05] than day 0 and 21 groups. The total protein content of the exercised mice group [0.93 ± 0.09] was significantly higher [p < 0.05] compared to the control [0.54 ± 0.16] and colostrum supplemented mice (0.64 ± 0.19), but did not differ significantly from the exercise with colostrum [0.78 ± 0.09] after 21 days of study period. The results of the Mauchly’s Test of Sphericity in the repeated analysis of variance procedure gave a p-value of less than 0.001. The Greenhouse-Geisser method was used to test for the time and time*group interaction effects. The results showed that the means for at least one pair of time points was different [F = 20.337, df = 1.455, p < 0.001, Eta square = 0.504, Power = 99.9.3%]. Also, there was a sizeable time*group interaction effect [F = 8.450, df = 4.366, p < 0.001, Eta square = 0.559, Power = 99.7%] [Table 1].

**Table 1:** Total Protein (mg/ml) after exercise and colostrum ingestion in mice

	**Control**	**Experimental groups**		
		**Colostrum**	**Exercise**	**Exercise + Colostrum**
Day 0	0.66 ± 0.14	0.63 ± 0.08	0.69 ± 0.13	0.51 ± 0.04
Day 21	0.54 ± 0.16	0.64 ± 0.19 ^⋆^	0.93 ± 0.09 ^⋆■ ●^	0.78 ± 0.09 ^⋆■♦^
Day 42	0.56 ± 0.05	1.09 ± 0.28 ^♦✞■^	1.06 ± 0.09 ^♦■✞●^	1.46 ± 0.50 ^⋆■✞♦^

Significance: ^⋆^ P < 0.05 – Significantly different from control groups; ^■^ P < 0.05 – Significantly different from ‘day 0’ group; ^✞^ P < 0.05 – Significantly different from ‘day 21’ group; ^●^ P < 0.05 – Significantly different from ‘colostrum’ group; ^♦^ P < 0.05 – Significantly different from ‘exercise’ group.

### Lipid hydroperoxides

The mean and standard deviation [SD] values for lipid peroxidation of skeletal muscle homogenate from mice in the four groups over time are shown in Table 2. Results of one way ANOVA analysis of data at baseline showed no differences between the groups at the beginning of the study. The mean for the exercise group was significantly higher [p < 0.05] compared to the other three groups at Day 21. There was a significant decrease in lipid hydroperoxides in colostrum alone group [p < 0.05] than the control group after 21 and 42 days. The means for the exercise plus colostrum group was significantly lower [p < 0.05] than the control group. At the end of the study period [Day 42], the mean LPO for the exercised mice group [6.4 ± 0.65] was significantly higher [p < 0.05] compared to the other three groups. The Mauchly's Test of Sphericity in the repeated analysis procedure gave a p-value of less than 0.001. The Greenhouse-Geisser method was used to test for time effect and time*group interaction effect. The results showed that the means for at least one pair of time points was different [F = 7.2, df = 1.8, P < 0.0001, Eta square = 0.27, Power = 89.4%]. Also, there was a sizeable time*group interaction [F = 36.95, df = 5.4, P < 0.001, partial eta square = 0.85, Power > 99.9%] [Table 2].

**Table 2:** Lipid hydroperoxides (nmol/ml/mg of protein) after exercise and colostrum ingestion in mice

	**Control**	**Experimental groups**		
		**Colostrum**	**Exercise**	**Exercise + Colostrum**
Day 0	3.75 ± 0.54	3.54 ± 0.44	3.63 ± 0.28	3.43 ± 0.17
Day 21	3.64 ± 0.59	2.88 ± 0.52 ^⋆■^	4.93 ± 0.18 ^⋆■ ●^	2.59 ± 0.28 ^⋆■♦^
Day 42	3.67 ± 0.47	2.41 ± 0.59 ^⋆■^	6.40 ± 0.65 ^⋆■✞●^	2.12 ± 0.48 ^⋆■✞♦^

Significance: ^⋆^ P < 0.05 – Significantly different from control groups; ^■^ P < 0.05 – Significantly different from ‘day 0’ group; ^✞^ P < 0.05 – Significantly different from ‘day 21’ group; ^●^ P < 0.05 – Significantly different from ‘colostrum’ group; ^♦^ P < 0.05 – Significantly different from ‘exercise’ group.

### Superoxide dismutase

The mean values obtained for SOD in the muscle homogenates of mice in various groups over time are shown in Table 3. Results of on one way analysis of data at baseline and after Day 21 showed that there were no significant differences in SOD values between all four groups. At day 42, the mean SOD value of exercised mice group [8.08 ± 0.66] was significantly lower [p < 0.05] than the other three groups. There was no difference in means between the colostrum and colostrum with exercise groups. A significant increase [p < 0.05] in muscle SOD was seen after colostrum ingestion with exercise and this increase was significantly higher than control groups [P < 0.05] after day 21 and 42. The Mauchly’s Test of Sphericity in the repeated analysis procedure gave a p-value of more than 0.05. The results showed that the means for at least one pair of time points was different [F = 4.857, df = 2, p = 0.013, Eta square = 0.195, Power = 77.1%]. Furthermore, a sizeable time*group interaction was observed [F = 2.282, df = 6, p = 0.055, partial Eta square = 0.255, Power = 72.73%] [Table 3].

**Table 3:** Superoxide Dismutase (U/ml/mg of protein) after exercise and colostrum ingestion in mice

	**Control**	**Experimental groups**		
		**Colostrum**	**Exercise**	**Exercise + Colostrum**
Day 0	12.46 ± 0.23	12.58 ± 0.23	12.11 ± 0.67	12.90 ± 0.64
Day 21	12.67 ± 0.87	14.55 ± 0.60 ^⋆■^	9.29 ± 0.40 ^⋆■●^	16.07 ± 0.75 ^⋆■♦^
Day 42	12.13 ± 0.86	14.65 ± 0.87 ^⋆■^	8.08 ± 0.66 ^⋆■✞●^	15.74 ± 0.35 ^⋆■✞♦^

Significance: ^⋆^ P < 0.05 – Significantly different from control groups; ^■^ P < 0.05 – Significantly different from ‘day 0’ group; ^✞^ P < 0.05 – Significantly different from ‘day 21’ group; ^●^ P < 0.05 – Significantly different from ‘colostrum’ group; ^♦^ P < 0.05 – Significantly different from ‘exercise’ group.

### Xanthine oxidase

The mean values obtained for xanthine oxidase in the skeletal muscle of mice for the various groups over time are shown in Table 4. The one way analysis of variance procedure on the data revealed that at baseline, there was no significant difference in the mean values between the four groups. After Day 21, the mean for the exercise alone group was significantly higher [p < 0.05] than the mean for the control, colostrum alone and colostrum with exercise groups; while, at Day 42, the mean for exercise group was again significantly higher [p < 0.05] than the mean of the other groups. Colostrum ingestion with exercise was able to reduce the xanthine oxidase level more significantly [p < 0.05] after 21 and 42 days. After 42 days of colostrum ingestion, there was a significant reduction in the muscle xanthine oxidase level which was significantly less [p < 0.05] than the control mice. The repeated ANOVA procedure was used to analyze the group and time factor interaction, and the Greenhouse-Geisser method was deployed to test for Time and Time*Group interaction effects. The Mauchly's Test of Sphericity revealed a p-value of less than 0.001. The results indicate that the means for at least one pair of time points was different [F = 13.212, df = 1.139, P < 0.001, partial eta square = 0.398, Power = 95.3%]. Also, there was a sizable time*group interaction [F = 3.694, df = 3.412, P < 0.05, partial eta square = 0.357, Power = 76.3%] [Table 4].

**Table 4:** Xanthine oxidase (μU/ml/mg of protein) after exercise and colostrum ingestion in mice

	**Control**	**Experimental groups**		
		**Colostrum**	**Exercise**	**Exercise + Colostrum**
Day 0	151.07 ± 2.62	150.23 ± 4.12	151.57 ± 2.07	151.59 ± 2.13
Day 21	151.67 ± 2.41	147.62 ± 2.86	176.74 ± 2.23 ^⋆■●^	144.62 ± 2.24 ^■♦^
Day 42	148.68 ± 3.86	146.76 ± 3.57	199.85 ± 2.18 ^⋆■✞●^	138.49 ± 2.51 ^⋆■✞●♦^

Significance: ^⋆^ P < 0.05 – Significantly different from control groups; ^■^ P < 0.05 – Significantly different from ‘day 0’ group; ^✞^ P < 0.05 – Significantly different from ‘day 21’ group; ^●^ P < 0.05 – Significantly different from ‘colostrum’ group; ^♦^ P < 0.05 – Significantly different from ‘exercise’ group.

### Total antioxidants

The means obtained for total antioxidants found in the muscle homogenate of mice from the various groups over time are shown in Table 5. There was no difference in the means between the four groups at baseline. After 21 days, the mean for the exercised mice group [0.39 ± 0.04] was significantly lower [p < 0.05] compared to the other three groups. The mean for the control group [1.03 ± 0.09] was significantly lower [p < 0.05] compared to the colostrum alone [1.42 ± 0.09] and exercise plus colostrum [1.47 ± 0.08] groups. Colostrum supplementation has significantly increased [p < 0.05] the total antioxidant level in skeletal muscle after 21 and 42 days in exercise with colostrum group and this increase was significantly higher [p < 0.05] than control and colostrum alone groups. The Mauchly's Test of Sphericity in the repeated ANOVA procedure had shown a p-value of less than 0.001. The Greenhouse-Geisser method used to test for time and time*group interaction effect showed that there was a moderate difference in means for at least one pair of time points [F = 3.603, df = 1.388, P = 0.056, partial eta square = 0.153, Power = 52.3%]. Also, there was a sizeable time*group interaction [F = 17.350, df = 4.165, P < 0.001, partial eta square = 0.722, Power > 99.9%] [Table 5].

**Table 5:** Total antioxidants (mM/ml/mg of protein) after exercise and colostrum ingestion in mice

	**Control**	**Experimental groups**		
		**Colostrum**	**Exercise**	**Exercise + Colostrum**
Day 0	0.97 ± 0.33	1.13 ± 0.15	0.92 ± 0.11	0.81 ± 0.06
Day 21	1.03 ± 0.09	1.42 ± 0.09 ^♦^	0.39 ± 0.04 ^⋆■●^	1.47 ± 0.08 ^⋆■♦^
Day 42	0.99 ± 0.23	1.56 ± 0.08 ^⋆■^	0.21 ± 0.09 ^⋆■✞●^	1.67 ± 0.06 ^⋆■✞♦^

Significance: ^⋆^ P < 0.05 — Significantly different from control groups; ^■^ P < 0.05 — Significantly different from ‘day 0’ group; ^✞^ P < 0.05 — Significantly different from ‘day 21’ group; ^●^ P < 0.05 — Significantly different from ‘colostrum’ group; ^♦^ P < 0.05 — Significantly different from ‘exercise’ group.

Benefits of physical exercise in disease prevention are well established. However, it has also been shown that heavy exercise increases the risk of oxidative damage to skeletal muscles due to accumulation of free radicals [13,16,17]. The risk of oxidative damage to skeletal muscle increases with aging due to increased rate of reactive oxygen species production in aged muscle during and after exercise. Increased oxidative stress provokes inflammatory muscle, damage further oxidative stress and muscular dysfunction [18,19]. Hence supplementation with antioxidants would be beneficial to reduce the skeletal muscle oxidative stress during regular muscular exercise to maintain normal muscle function. Antioxidants are free radical scavenging molecules that prevent the cellular damage by oxidative stress and prevent the disease processes. There are many antioxidants within the organ systems that protect the critical cellular structures against the damage from oxygen free radicals and the products of lipid peroxidation. Catalase, superoxide dismutase, and glutathione peroxidase are the important non-enzymatic antioxidants that quench the free radicals and will increase the release of other antioxidants in the organ system [19-21].

Bovine colostrum is the first milk produced by cows and it contains various bioactive compounds that provide a strong nutritional support for the newborn. BC is currently promoted as a supplement in sports nutrition for muscle recovery, anaerobic sports functions and also to some extend as anti-aging property [3,4]. The results of the present study showed that there was a main effect of time and significant impact on antioxidant levels including, lipid peroxidation [LPO], superoxide dismutases [SODs], xanthine oxidase [XO] and total antioxidant [TO], all of which indicate a positive influence following colostrum supplementation. There are studies reporting that antioxidants are incapable of extending exercise-induced lifespan extension in rats [22]. Exercise-induced reactive oxygen species itself are known to increase endogenous reactive oxygen species defense capacity in skeletal muscles [22,23] and antioxidant supply may prevent the induction of molecular regulators of endogenous antioxidant defense in the skeletal muscles during exercise. But the results of this study showed that the exercise group had increased levels of oxidative stress. Moderate levels of reactive oxygen species in any tissue are necessary for the normal homeostatic process but excessive production causes oxidative stress. With increased intensity and prolonged exercise, a skeletal muscle mitohormesis process might have been suppressed to cause increased oxidative stress. Accumulated reactive oxygen species during prolonged exercise might have enhanced oxidative damage in skeletal muscles as the muscle proteins are highly redox sensitive [24-26].

The exercised group showed positive results in the present study, which concurs with previous studies that exercise certainly had a significant role in inducing oxidative stress. Antioxidant supplementation had also shown a significant impact on lipid peroxidation in highly trained athletes or over-trained athletes, and also had helped to increase the antioxidants level in intermittent players [19,26-29].

Chiang and Chang [30] reported antioxidant properties of caseins and whey proteins in colostrum. Colostrum contains significantly higher quantities of antioxidants which is very crucial for health to protect against oxidative stress in infants. It is known that at birth, the newborns will go through many challenges and one such challenge is adaptation to the oxygen-rich environment compared to the low-oxygen intrauterine environment [31-33]. However, this situation may be overcome by the generation of excessive reactive oxygen species [ROS]. Colostrinin [CLN] is a complex of proline rich polypeptide derived from bovine colostrum, which induces mitogenic stimulation as well as cytokines in human peripheral blood leucocytes and it also possesses antioxidant activity in pheochromocytoma [PC1] cells [1,31,34].

Our results showed that there was severe oxidative stress after exercise in mice which was supported by an increased level of lipid hydroperoxides and xanthine oxidase and a reduced total antioxidants and superoxide dismutase. Observed severe oxidative stress in skeletal muscles after exercise is in agreement with earlier reports. Many studies have reported that exercise increases free radicals accumulation in skeletal muscles and this will result in increased release of the antioxidant enzymes into circulation [21,22,35]. There will be increased formation of protein carbonyl compounds which are the markers of lipid peroxidation indicating severe oxidative stress within skeletal muscles [36]. Few studies have shown that higher concentrations of antioxidants instead of protecting skeletal muscles against oxidative stress, increases oxidative damage during exercise. Higher concentrations of antioxidants are reported to eliminate the reactive oxygen species which normally play an important role in the regulation of redox sensitive muscle proteins during muscle contractions [26,27]. But the results of this study demonstrated that concurrent colostrum treatment was able to improve the total antioxidant levels and increase the skeletal muscle superoxide dismutase level and reduce the lipid hydroperoxide and xanthine oxidase formation. These results demonstrated that bovine colostrum may have a significant antioxidant effect in skeletal muscle after muscular exercise. Several earlier studies have reported the use of bovine colostrum for immune responses and improved sporting performances [8,11,37]. But there are no reports about the effects of bovine colostrum in modulating the skeletal muscle antioxidant defense system. Bovine colostrum has significant amounts of enzymatic and non-enzymatic antioxidants. Lactoperoxidase, catalase, superoxide dismutase and glutathione peroxidase are the important enzymatic antioxidants present in bovine colostrum. These colostrum antioxidants were able to quench the exercise-induced free radicals in skeletal muscle [31,37].

In majority of the studies supporting the detrimental effects of antioxidant supplement during exercise, the antioxidants tested are Vitamin C, E and Coenzyme Q10 [24,27]. The observed effects could be due to lack of proper experimental design or dosage and duration of the treatment. Whereas in the present study, mice with or without exercise were force fed with colostrum throughout the duration of the experiment, which has shown the beneficial effects of this supplement during the exercise. Thus, the type of antioxidant and the dose and duration is likely to be a significant factor in protecting skeletal muscle against prolonged exercise –induced oxidative damage.

## Conclusions

In conclusion, the study has confirmed the antioxidant potential of bovine colostrum in muscular exercise. Bovine colostrum supplementation was able to decrease skeletal muscle lipid hydroperoxides and xanthine oxidase and increase in superoxide dismutase. Colostrum as an antioxidant supplementation increased the antioxidant reserve and protects skeletal muscle against exercise-induced oxidative injury. Further research is being undertaken to look into the molecular mechanism of colostrum induced antioxidant effects in skeletal muscles during exercise.

## Methods

### Animals

Adult male BALB/c mice [n = 72, 30–40 g] were obtained from University Sains Malaysia, Kelantan, Malaysia. Mice were acclimatized to the housing facility and allowed free access to water and feed [LabDiet, USA] for one week before the start of the experiment. The study was conducted according to the guidelines laid down in the declaration of Helsinki and all the experimental procedures are in accordance with the ARRIVE (Animal Research Reporting In Vivo Experiments) guidelines. All the procedures involving experimental animals were approved by the Institutional Research and Ethics Committees of International Medical University (IMU), Malaysia and University Technology MARA (UITM), Malaysia.

### Diet and experimental groups

Commercially available immunoglobulins enriched bovine colostrum provided by SmartNaco Sdn Bhd and Promec Sdn Bhd, Malaysia was used in this study. The composition of the colostrum and the normal rodent diet used in the study is shown in Table 6. The mice were randomly divided into four groups (Control-vehicle, Control-colostrum, Exercise-vehicle, and Exercise + colostrum) and each group had three subgroups [day 0, day 21 and day 42]. Initial body weights did not vary between different groups. Control groups of mice had access to free rodent diet and water throughout the duration of the experiment. The colostrum group received 0.1 ml volume of bovine colostrum [50 mg/day/mouse] by oral gavage. The exercise group of mice were made to exercise on the treadmill for 30 minutes per day. The fourth group of mice were made to exercise on the treadmill daily for 30 minutes and were fed daily with bovine colostrum [Table 7]. At the end of the experiment, the mice were weighed and killed by over dose of anaesthesia [sodium pentobarbital, 50 mg/kg body weight, intraperitoneal injection].

**Table 6:** Composition of rodent diet and bovine colostrum

	**Basal diet (/100 g)**	**Colostrum (w/w)**
Carbohydrate	57.9 g	6%
Protein	23.9 g	84.7%
Fat	10.7 g	1.8%
Total calories	4.07 kcal	60.0 kcal

**Table 7:** The study groups

**Control**	**Experimental groups**		
	**Colostrum**	**Exercise**	**Exercise + Colostrum**
Day 0	Day 0	Day 0	Day 0
Day 21	Day 21	Day 21	Day 21
Day 42	Day 42	Day 42	Day 42

### Exercise procedure

The exercise protocol in this study consisted of treadmill running [IITC, Model: 800, USA]. This mode of exercise was used because exercise intensity and duration could be experimentally manipulated and quantified, unlike voluntary wheels or swimming. Prior to the start of the study, the mice were allowed to familiarize with running on the treadmill for three days. During the familiarization period, the mice were made to run on the treadmill for approximately 10 – 15 minutes per day at a speed of 8 m/min. The mice were made to exercise on the treadmill for 30 minutes, between 10 AM and 12 AM daily for three weeks and six weeks in different groups.

After 24 h after the last day of the experimental duration, the body weights of the animals were recorded and the mice were sacrificed by an overdose of anaesthesia. The vastus lateralis muscles from the mouse hind legs were removed and placed into a petri dish containing 3 ml of cold phosphate buffer. The muscle tissues were fragmented into small pieces and minced into a paste like tissue using fine scissors. Minced muscle tissues were transferred into a 50 ml tube and the mixture was mechanically homogenised using a hand held cell homogeniser [Wiggen Hauser, Germany] at 9000 rpm for 5 min and the homogenisation process was repeated for 4 cycles. The homogenised sample was allowed to settle in ice for 2–5 min and then filtered using 70 μm and 100 μm cell strainers to exclude the unwanted cell debris and connective tissues. The filtered mixture was centrifuged at 2000 rpm for 10 min at 4°C and the supernatant was collected in a 15 ml tube and stored at −80°C before the ELISA analysis. Oxidative stress parameters like, total antioxidant, xanthine oxidase [XO], superoxide dismutase [SOD] and lipid hydroperoxides determination in the skeletal muscle homogenate were determined using commercially available assay kits according to the manufacturer’s protocol [Cayman Chemical, Ann Arbor USA].

### Lipid hydroperoxide assay

The muscle tissue homogenate stored at −80°C were thawed and for the sample preparation, an aliquot 500 μl of the sample supernatant was transferred into a glass test tube. Then an equal volume of Extract R of saturated methanol was added to each tube and vortexed for 1 minute. Later, 1 ml of cold chloroform was added to each tube and mixed thoroughly using vortex. The mixture was centrifuged at 1,500 x g for 5 minutes at 0°C. Then the bottom chloroform layer was collected by inserting a Pasteur pipette along the side of the test tube. This layer was then transferred to another test tube and stored in ice. At all times, it was ensured that no water was mixed with the procedures to avoid interference with colour development.

Later, 500 μl of the chloroform extract of each sample was added to appropriately labelled glass test tubes. Water needs to be avoided at all times from the extract. Next, an aliquot 450 μl of chloroform-methanol solvent mixture was added to the sample test tubes. Then, the chromogen was prepared by mixing equal volumes of FTS reagent 1 and FTS reagent 2 in a test tube and mixed thoroughly using vortex. 50 μl of the freshly prepared chromogen was added to each assay tube and mixed well by vortexing.

Next, the test tubes were close with caps and incubated for five minutes at room temperature. Immediately after 5 minutes, 300 μl of the incubated assay tubes were transferred into 96-well plate. Subsequently, the absorbance was read at 500 nm using a 96-well plate reader. The average absorbance of each standard and sample was calculated. The graph was plotted using the absorbance and standard. The hydroperoxide values [HP] of the sample tubes were calculated using the equation [HP [nmol] = [sample absorbance – y-intercept] / slope] obtained from the linear regression of the standard curve substituting corrected absorbance values for each sample.

### Superoxide dismutase assay

The homogenate from the skeletal muscle tissue stored at −80°C were thawed and used for the sample preparation. Add 200 μl of the diluted radical detector and 10 μl of standard per well for the standard wells. Next in the sample wells, 200 μl of the diluted radical detector and 10 μl of sample were added to the wells. Subsequently, the reaction was initiated by adding 20 μl of diluted xanthine oxidase to all the wells. The 96-well plates were gently shaken for 2 seconds to mix and then covered with the plate cover. The plate was incubated on a shaker for 20 minutes at room temperature. Thereafter, absorbance was read at 450 nm using a plate reader. The mean absorbance was calculated for each standard and sample. The linearized SOD standard rate [LR] as a function of final C was plotted. Next, the SOD activity using the equation form the linear regression of the standard curve substituting the linearized rate [LR] for each sample was calculated.

### Xanthine oxidase assay

Firstly, the standard wells were prepared by adding 50 μl of XO Standard. The supernatants from muscle tissue supernatant stored at -80°C were thawed and used for the sample preparation. Later, 50 μl of the sample supernatant was added into the test wells in duplicate samples. A 50 μl of Assay Cocktail [supplied by the supplier] was added to all wells. Then, the plate was covered with a plate cover and incubated for 45 minutes at 37°C. The fluorescence was read using an excitation of wavelength of 500 nm and an emission of 590 nm. The mean fluorescence of each standard and sample was then calculated. The fluorescence values of each standard were plotted. The xanthine oxidase concentration of the samples using the equation of the linear regression of the standard curve was calculated.

### Total antioxidant assay

The assay standard was prepared according to the supplier instructions. Add the 10 μl of trolox standard, 10 μl metmyoglobin and 150 μl of chromogen per well in the standard designated well in the 96-well plate. After thawing the muscle supernatant sample, 10 μl of sample, 10 μl of metmyoglobin and 150 μl of chromogen was added into the test wells in duplicate samples. Later, 40 μl of hydrogen peroxide was added to initiate the reactions. Immediately after that, the plate was covered and incubated on a shaker for 5 minutes at room temperature. The absorbance was read at 750 nm or 405 nm using a plate reader. The mean absorbance was calculated for each standard and sample. The average absorbance of standards was plotted. Total antioxidant concentration of the sample was calculated using the linear regression equation. The assay range was between 0.044–0.330 mM.

### Total protein assay

The Bradford protein determination method was used in determining the total protein in the muscle homogenates. Firstly, the standard for the protein determination following the manufacturer’s instructions [Cayman Chemical, Ann Arbor USA] was prepared and then 100 μl of standard was added into standard wells as per instruction. 100 μl of sample was added into the test wells in duplicate samples. Later, 100 μl of assay reagent was added to each well. The plate was incubated for 5 minutes at room temperature. After the incubation period, absorbance was read at 595 nm using a plate reader. The graph was plotted using the mean absorbance of the standards and the concentration by linear regression formula was estimated.

### Statistical analysis

Data were analyzed using SPSS for Windows Version 17. Descriptive data were generated for all variables and presented as means ± standard deviation [SD]. One way analysis of variance [ANOVA] was used to determine the significance differences between control and experimental animals. When analysis of variance indicated significant differences, post-hoc testing of differences between groups was performed using Bonferroni test. The effect size of parameters was presented to observe the effect of the treatment versus the control using ANOVA test. A value of p < 0.05 was considered as statistically significant.

## Competing interest

The authors declare that there are no competing interests

## Authors’ contributions

MA, AKR and NH carried out the experimental work and drafted the manuscript. KR and RR helped in the animal work and biochemical analysis. ABAM, KR, MNI, NS conceived the study, and participated in its design and coordination and helped to draft the manuscript. KC and NH helped in the statistical analysis. All authors read and approved the final manuscript.
